# Molecular and Genetic Determinants of the NMDA Receptor for Superior Learning and Memory Functions

**DOI:** 10.1371/journal.pone.0111865

**Published:** 2014-10-31

**Authors:** Stephanie Jacobs, Zhenzhong Cui, Ruiben Feng, Huimin Wang, Deheng Wang, Joe Z. Tsien

**Affiliations:** 1 Brain and Behavior Discovery Institute and Department of Neurology, Medical College of Georgia at Georgia Regents University, Augusta, Georgia, United States of America; 2 Shanghai Institute of Functional Genomics, East China Normal University, Shanghai, China; 3 Banna Biomedical Research Institute, Xi-Shuang-Ban-Na Prefecture, Yunnan Province, China; Louisiana State University Health Sciences Center, United States of America

## Abstract

The opening-duration of the NMDA receptors implements Hebb's synaptic coincidence-detection and is long thought to be the rate-limiting factor underlying superior memory. Here, we investigate the molecular and genetic determinants of the NMDA receptors by testing the “synaptic coincidence-detection time-duration” hypothesis vs. “GluN2B intracellular signaling domain” hypothesis. Accordingly, we generated a series of GluN2A, GluN2B, and GluN2D chimeric subunit transgenic mice in which C-terminal intracellular domains were systematically swapped and overexpressed in the forebrain excitatory neurons. The data presented in the present study supports the second hypothesis, the “GluN2B intracellular signaling domain” hypothesis. Surprisingly, we found that the voltage-gated channel opening-durations through either GluN2A or GluN2B are sufficient and their temporal differences are marginal. In contrast, the C-terminal intracellular domain of the GluN2B subunit is necessary and sufficient for superior performances in long-term novel object recognition and cued fear memories and superior flexibility in fear extinction. Intriguingly, memory enhancement correlates with enhanced long-term potentiation in the 10–100 Hz range while requiring intact long-term depression capacity at the 1–5 Hz range.

## Introduction

N-methyl-D-aspartate (NMDA) receptors are known to be the key modulators of synaptic plasticity in the forebrain regions [1]–[4] and act as the molecular gating switch for learning and memory [5], [6]. It is widely accepted that their unique coincidence detection property allows them to impart Hebb's rule on synapses, by requiring the simultaneous pre-synaptic release of glutamate and the depolarization of the postsynaptic membrane to remove the extracellular Mg^2+^ block [7]. NMDA receptors are composed of two GluN1 subunits, as well as two GluN2 subunits [8]. In the adult forebrain regions, GluN2A and GluN2B subunits are the main subunits available in excitatory synapses for receptor complex formation [8], [9], and are ideal for coincidence detection due to their strong Mg^2+^ dependency [10]–[12].

During postnatal brain development, the GluN2B subunits are the predominate subunits expressed specifically in excitatory neurons of the forebrain regions, such as the cortex and hippocampus [9], [13]. As the animal develops into adulthood, GluN2B expression decreases while GluN2A expression increases, resulting in an overall decrease in synaptic plasticity. Previously, we have shown that an overexpression of the GluN2B subunit in the forebrain excitatory neurons enhances many forms of learning and memory in both transgenic mice and rats [14]–[18]. The prevalent view in the field is that memory enhancement by GluN2B up-regulation is due to its longer opening duration in comparison to that of the GluN2A subunit. This enables the GluN2B-containing NMDA receptors to be better coincidence detectors.

However, the different structural motifs of GluN2 subunits are known to regulate the NMDA receptors' Mg^2+^ dependency, channel opening duration, magnitude of Ca^2+^ influx, as well as, intracellular signaling cascades [10]. For example, the GluN2A and GluN2B subunits have high Mg^2+^ dependency, whereas the GluN2C and GluN2D subunits have much less Mg^2+^ dependency [13]. Thus, the extracellular Mg^2+^ blockade of the GluN2A or GluN2B-containing NMDA receptors suppresses NMDA-mediated Ca^2+^ influx at voltages close to the resting membrane potential allowing the cell to differentiate between correlated synaptic input and uncorrelated activity [7], [19], [20]. Recent studies have further demonstrated that the C-terminals of GluN2A and GluN2B bind to different downstream signaling molecules. This has led to a greater appreciation for their contribution to synaptic plasticity and behavior [21], [22]. To examine the effects of GluN2A on learning and memory, we recently generated CaMKII promoter-driven GluN2A transgenic mice and found profound long-term memory deficits in these mice, while their short-term memories remain unaffected [23]. Therefore, overexpression of GluN2A or GluN2B in the mouse forebrain leads to impaired or enhanced memory function, respectively. These observations have raised several key questions, as to whether enhanced memories in GluN2B transgenic mice or impaired memories in GluN2A transgenic mice were due to their differences in NMDA receptor channel-opening durations or their distinct intracellular signaling processes. Answers to this crucial question can be highly valuable for developing therapeutic strategies for preventing memory loss in patients.

Currently, two hypotheses have been postulated to explain the observed memory enhancement in the GluN2B transgenic animals or memory impairment in GluN2A transgenic mice [4]. One hypothesis, known as the “coincidence-detection” hypothesis, posits that because the GluN2B subunit makes the channel opening duration longer than that of the GluN2A subunit, the GluN2B overexpression allows a greater coincidence detection window, thereby leading to superior memory functions. The shorter coincidence-detection, such as in the GluN2A transgenic mice, underlies impaired long-term memories. The second hypothesis is that the distinct intracellular domain of the GluN2B subunit is responsible for the enhancements observed in the GluN2B transgenic mice [4]. Several recent key observations support this “intracellular domain hypothesis”. Biochemical studies have shown that the intracellular C-terminal domains of the GluN2A and GluN2B subunits preferentially interact with different downstream molecules and play distinct roles in synaptic functions [24]. Conversely, studies using genetically truncated GluN2A or GluN2B subunits demonstrate that the C-terminal connections are essential for NMDA receptor function. The truncated subunits often act as functional knockouts of the whole subunit [21]. Although several truncated C-terminal studies have focused on the mechanisms by which the GluN2 subunits mediate the NMDA receptor functions, the structural motifs crucial for learning and memory enhancement remain undefined. It is completely unknown as to whether and what degree the C-terminal domain of the GluN2B would contribute to memory enhancement.

In the present study, we set out to examine the above two hypotheses aimed at determining how and whether the molecular motifs underlying coincidence-detection time duration, or intracellular signaling cascades play a role in enhancing learning and memory. Our strategy is to swap or replace the C-terminal cytoplasmic domain of the GluN2B subunit with the C-terminal domain of the GluN2A subunit, or vice versa. Additionally, we have replaced the C-terminal domain of the GluN2D subunit with the C-terminal domain of the GluN2B subunit effectively reducing the Mg^2+^ dependency of the receptor. We have produced and analyzed five different GluN2 transgenic mouse lines, namely, GluN2A transgenic mice (Tg-GluN2A), Tg-GluN2B^2A(CT)^ transgenic mice, GluN2B transgenic mice (Tg-GluN2B), Tg-GluN2A^2B(CT)^ transgenic mice, and Tg-GluN2D^2B(CT)^ transgenic mice. Our experiments suggest that the C-terminal domain of the GluN2B subunit is necessary and sufficient to produce memory enhancement, as long as it is coupled to Mg^2+^ dependent forms of GluN2 subunits such as GluN2A or GluN2B, while coupling of the GluN2A's C-terminal domain to GluN2B N-terminal and transmembrane domains lead to profound memory impairment. Moreover, coupling of the C-terminal domain of the GluN2B subunit to GluN2D subunit's N-terminal and transmembrane domains lead to memory deficits.

## Results

### Generation of transgenic mice expressing chimeric GluN2A^2B(CTR)^, GluN2B^2A(CTR)^, or GluN2D^2B(CTR)^ subunits in the forebrain principal neurons

**Figure 1:**
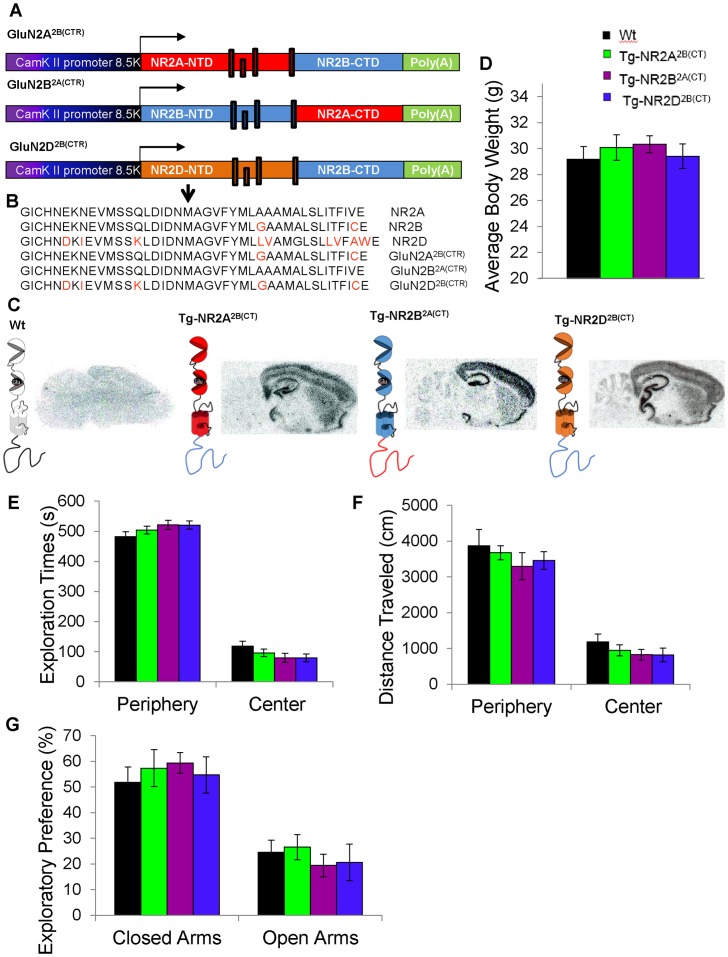
Constructs and basic behavioral assays of the GluN2 chimeric mice. (**A**) Illustration of the constructs used to create the GluN2A^2B(CTR)^, GluN2B^2A(CTR)^, and GluN2D^2B(CTR)^ chimeric subunits. (**B**) A point mutation was made on the cloning vector to induce an Aat II cutting sites to link the N-terminal and membrane domain to the C-terminal domain. After successfully joining the domains, the point mutation was restored to the original sequence. The arrow indicates the fusion position located in trans-membrane domain. (**C**) *In situ* hybridization of the transgene expression in the wild-type mice (Wt), the Tg-GluN2A^2B(CT)^ mice, the Tg-GluN2B^2A(CT)^ mice and the Tg-GluN2D^2B(CT)^ mice using SV-40 probes with a schematic of the receptor subunit expressed in the excitatory neurons. (**D**) No differences were found in the average adult body weight of the wild-type mice, the Tg-GluN2A^2B(CT)^, Tg-GluN2B^2A(CT)^, and Tg-GluN2D^2B(CT)^mice. (**E**) The chimeric transgenic mice spent similar amounts of time as the wild-type mice in the center verses the periphery of the open field arena. (**F**) The chimeric transgenic mice and the wild-type mice showed similar locomotion in the open field. (**G**) The Tg-GluN2A^2B(CT)^, Tg-GluN2B^2A(CT)^ and Tg-GluN2D^2B(CT)^ mice spent similar amounts of time in the closed arms and the open arms of the elevated plus maze as the wild-type mice.

To investigate the potentially distinct roles of the C-terminal domains vs. the N-terminal and membrane domains in GluN2 subunits in mediating memory enhancement, we created constructs encoding chimeric receptors based on GluN2B and GluN2A but with their respective CTDs replaced (denoted as CTR) with each other's (GluN2B^2A(CTR)^ and GluN2A^2B(CTR)^, respectively. We have created three new chimeric GluN2 transgenic mouse lines. We used the same αCaMKII promoter for driving transgene expression in forebrain excitatory neurons as we did for producing the GluN2B [16] and GluN2A transgenic mice [23]. In the first transgenic line, termed Tg-GluN2B^2A(CT)^ chimeric transgenic mice, the C-terminal domain of the GluN2B subunit has been swapped for the counterpart C-terminal domain of the GluN2A and overexpressed in the forebrain excitatory neurons (Figure 1A). This effectively pairs the opening duration of the GluN2B subunit with the signaling domain of the GluN2A subunit. In the second transgenic line, termed Tg-GluN2A^2B(CT)^, the C-terminal domain of the GluN2A subunit has been swapped for the counterpart C-terminal domain of the GluN2B (Figure 1A). This chimeric subunit possesses the GluN2A opening duration but with the signaling domain from the GluN2B subunit. Additionally, to investigate the requirement of the Mg^2+^ dependent synaptic coincidence-detection function for producing GluN2B-mediated intracellular signaling, we created a third transgenic mouse line, namely, Tg-GluN2D^2B(CT)^ mice, in which the C-terminal domain of the GluN2B subunit has been fused to the N-terminal and membrane domain of the GluN2D subunit (which is less Mg^2+^-dependent) denoted as GluN2D^2B(CTR)^ (Figure 1A).

We confirmed the transgene integration into the genome of their off-spring by Southern Blot analysis using Poly(A) probes (Figure S1A) and Western Blot (Figure S1B). Next, we performed a series of *in situ* hybridization experiments to determine the expression pattern of the transgenes in the mouse brains. As shown, the transgenes are highly enriched in the cortex, striatum, and hippocampus, but not in hindbrain regions such as the cerebellum (Figure 1C). The high expression transgenic mice were crossed with C57BL/6J wild-type mice for at least 8 generations. These chimeric GluN2 transgenic offspring were found to grow and breed normally, having similar adult weights to their wild-type littermates (Figure 1D) (Wt: n = 11, 29.18±0.985 g; Tg-GluN2A^2B(CT)^: n = 7, 30.09±0.990 g; Tg-GluN2B^2A(CT)^: n = 10, 30.34±0.648 g; Tg-GluN2D^2B(CT)^: n = 9, 29.41±0.940 g), and being visually indistinguishable among them. Additionally, we also produced transgenic GluN2A overexpression mice (Tg-GluN2A mice) [23] and transgenic GluN2B overexpression mice (Tg-GluN2B mice) [14]–[17] for comparisons on learning and memory tests. These two transgenic mouse lines were also maintained on the same genetic background.

The Tg-GluN2A^2B(CT)^, Tg-GluN2B^2A(CT)^, and Tg-GluN2D^2B(CT)^ mice showed no differences in the open field behavioral paradigm, either in time spent in the center verses the periphery (Figure 1E) (center: Wt: n = 7, 117.91±17.006 s; Tg-GluN2A^2B(CT)^: n = 6, 95.95±12.893 s; Tg-GluN2B^2A(CT)^: n = 5, 78.74±15.154 s; Tg-GluN2D^2B(CT)^: n = 6, 79.15±13.483 s; periphery: Wt: 481.86±16.994 s; Tg-GluN2A^2B(CT)^: 503.98±12.901 s Tg-GluN2B^2A(CT)^: 520.96±15.098 s; Tg-GluN2D^2B(CT)^: 520.65±13.527 s), or in locomotor activity (Figure 1F) (center: Wt: 1184.87±218.32 cm; Tg-GluN2A^2B(CT)^: 945.79±158.233 cm; Tg-GluN2B^2A(CT)^: 824.15±147.123 cm; Tg-GluN2D^2B(CT)^: 819.96±195.070 cm; periphery: Wt: 3864.83±460.03 cm; Tg-GluN2A^2B(CT)^: 3675.25±196.950 cm; Tg-GluN2B^2A(CT)^: 3294.38±378.288 cm; Tg-GluN2D^2B(CT)^: 3458.34±249.227 cm). This suggests that these transgenic mice were normal in locomotor activity and anxiety. Additionally, no differences were found in the elevated plus maze paradigm, which also measured for anxiety-like behavior (Figure 1G). Therefore, the chimeric GluN2 mice were indistinguishable from their wild-type littermates in growth, body weights, and these basic behaviors.

### Enhancement of long-term object recognition memory in Tg-GluN2A^2B(CT)^ mice but impairments in long-term memory of the Tg-GluN2B^2A(CT)^ and Tg-GluN2D^2B(CT)^ mice

**Figure 2:**
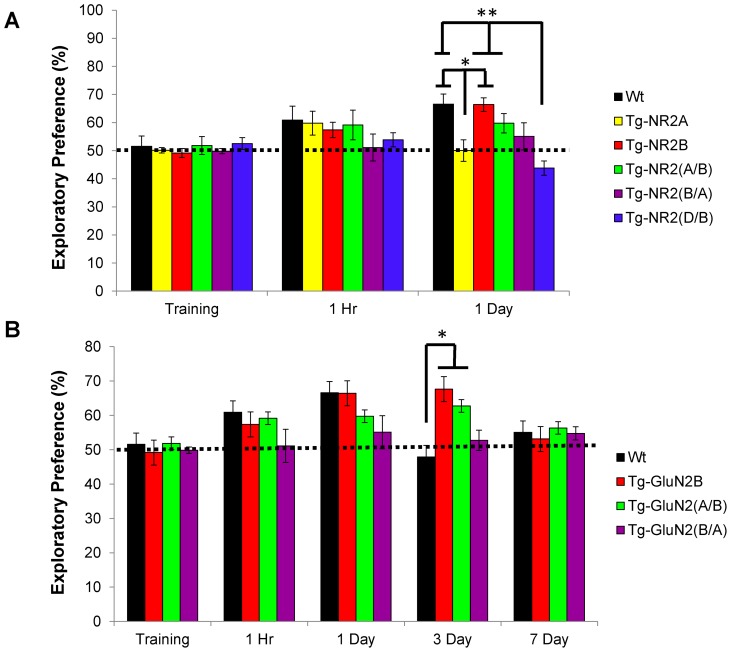
Enhanced long-term recognition memory of the Tg-GluN2A^2B(CT)^ mice and impaired long-term memory on the Tg-GluN2D^2B(CT)^ mice. (**A**) All groups of mice tested showed similar exploratory behavior in the training session. At the one hour retention session, the Tg-GluN2A, Tg-GluN2B and Tg-GluN2A^2B(CT)^ mice showed similar interest in the novel object as the wild-type mice. Whereas the Tg-GluN2B^2A(CT)^ and Tg-GluN2D^2B(CT)^ mice show almost no preference for the novel object. At the 24 hour retention test, as expected the Tg-GluN2A and Tg-GluN2D^2B(CT)^ mice showed no preference for the novel object. The Tg-GluN2B, The Tg-GluN2A^2B(CT)^ and Tg-GluN2B^2A(CT)^ mice all spent similar amounts of time with the novel object. *p = 0.003, **p = 7.7×10^−5^. (**B**) In addition to the enhancement seen in the Tg-GluN2A^2B(CT)^ mice at the 24 hour recall session, these mice also showed enhanced recognition memory even at 3 days post-training over the wild-type mice. *p = 0.003. Whereas the GLUN2A^2B(CT)^ mice show no preference for the novel object at 3 day or 7 days.

To investigate recognition memory functions in the transgenic mice, we tested the mice in a novel object recognition task for both short-term and long-term memory domains. During training, all transgenic mouse groups showed comparable exploratory behavior and motivation for the task, exploring each object to a similar degree (Figure 2A) (Wt: n = 10, 51.55±3.65%; Tg-GluN2A: n = 10, 50.15±0.932%; Tg-GluN2B: n = 9, 49.17±1.611%; Tg-GluN2A^2B(CT)^: n  = 10, 51.85±3.192%; Tg-GluN2B^2A(CT)^: n = 10, 49.85±0.932%; Tg-GluN2D^2B(CT)^: n = 20, 52.52±2.097%).

At the one hour retention session, the Tg-GluN2A, Tg-GluN2B and Tg-GluN2A^2B(CT)^ mice showed similar interest in the novel object as compared to the wild-type mice (Wt: n = 10, 60.90±4.913%; Tg-GluN2A: n = 11, 59.80±4.270%; Tg-GluN2B: n = 7, 57.38±2.76%; Tg-GluN2A^2B(CT)^: n = 14, 59.15±5.294%) demonstrating no changes in short-term recognition memory. Whereas the Tg-GluN2B^2A(CT)^ and Tg-GluN2D^2B(CT)^ mice show no preference for the novel object (Tg-GluN2B^2A(CT)^: n = 12, 51.15±4.808%; Tg-GluN2D^2B(CT)^: n = 22, 53.85±2.535%), suggesting memory impairment in this test.

At the 24 hour retention session, the Tg-GluN2A mice showed no preference for the novel object and significantly less interest in it than the wild-type mice (Figure 2A) (GluN2A: n = 10, 50.03±3.860%; *F*(2, 24)  = 7.45, p = 0.003) as noted previously [23], demonstrating their inability to form a long-term recognition memory. As expected, the Tg-GluN2D^2B(CT)^ mice also showed no preference for the novel object at the 24 hour retention session spending significantly less time exploring the novel object than the wild-type mice (Wt: n = 10, 66.56±3.610%; Tg-GluN2D^2B(CT)^: n = 20, 43.80±2.566%; *F*(5, 63)  = 6.36, p = 7.7×10^−5^). The Tg-GluN2B^2A(CT)^ mice spent only slightly more time with the novel object than the familiar object (n = 12, 55.10±4.821%). However, the Tg-GluN2B and Tg-GluN2A^2B(CT)^ mice showed similar memory of the novel object to the wild-type mice (Tg-GluN2B: n = 7, 66.44±2.417%; Tg-GluN2A^2B(CT)^: n = 14, 59.77±3.418%). This demonstrates impaired long-term recognition memory in the Tg-GluN2D^2B(CT)^ mice.

To determine the extent of the enhancement in the Tg-GluN2A^2B(CT)^ mice, separate cohorts of mice were further used to test in their ability to retain the memory of the object over three-day and seven-day periods. Remarkably, at the three day retention tests, the Tg-GluN2A^2B(CT)^ mice, like the Tg-GluN2B mice, spent significantly more time investigating the novel object (Figure 2B) (Tg-GluN2B: n = 7, 67.64±4.337%; Tg-GluN2A^2B(CT)^: n = 14, 62.76±2.968%) than the wild-type mice (Wt: n = 10, 47.93±4.045%, *F*(2, 28)  = 7.05, p = 0.003). However, the Tg-GluN2B^2A(CT)^ mice showed no interest in the novel object, spending approximately equal time with both objects (Tg-GluN2B^2A(CT)^: n = 8, 52.74±2.924%). At the seven day retention session, the Wt, GluN2B, Tg-GluN2B^2A(CT)^, and Tg-GluN2A^2B(CT)^ mice spent similar amounts of time investigating the novel object (Wt: n = 10, 55.05±4.096%; Tg-GluN2B: n = 6, 53.12±3.373%; Tg-GluN2A^2B(CT)^: n = 14, 56.36±3.344%; Tg-GluN2B^2A(CT)^: n = 8, 54.75±1.928%; *F*(2,35)  = 0.21, p = 0.93). This demonstrates the significant enhancement in long-term recognition memory in the Tg-GluN2A^2B(CT)^ mice, to a similar degree as the Tg-GluN2B mice did.

### Normal contextual fear memory in the chimeric transgenic mice

**Figure 3:**
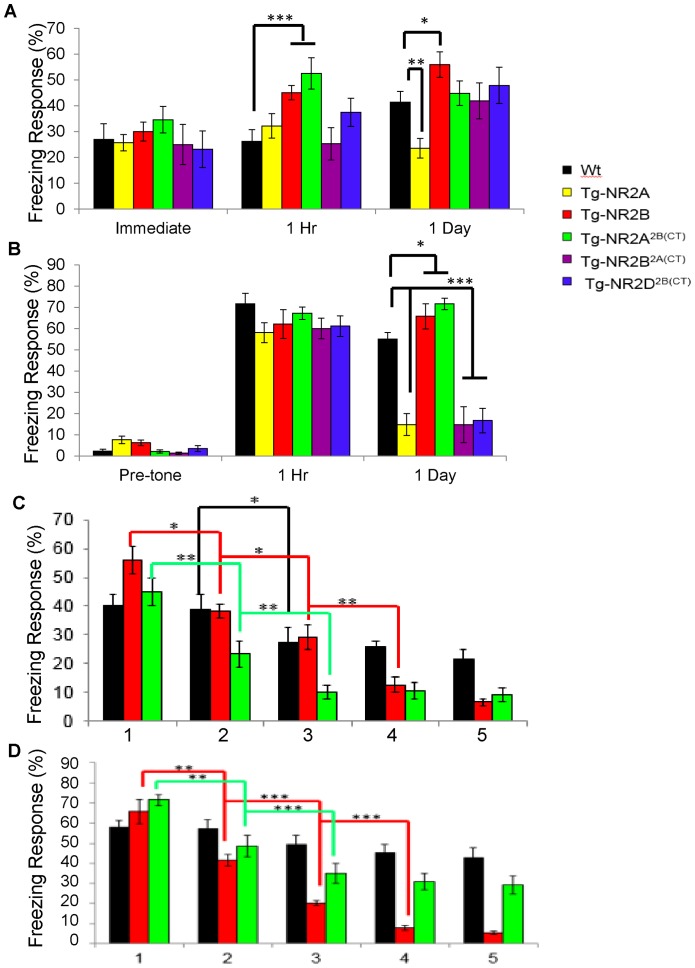
Selectively impaired emotional memory in the Tg-GluN2B^2A(CT)^ and Tg-GluN2D^2B(CT)^ mice. (**A**) The mice showed similar freezing responses immediately following the US. At the one hour retention session, the wild-type mice, the Tg-GluN2A, Tg-GluN2B^2A(CT)^ and Tg-GluN2D^2B(CT)^ mice all displayed similar freezing responses. Interestingly, both the Tg-GluN2B and the Tg-GluN2A^2B(CT)^ mice spent significantly more time freezing. At the 24 hour recall session only the Tg-GluN2A mice demonstrated a diminished freezing response to the context in which the shock was delivered. *p = 0.005, **p = 0.0007. (**B**) The mice tested also showed similar pre-tone freezing responses and similar freezing at the one hour contextual recall. At the 24 hour recall session the Tg-GluN2A mice, the Tg-GluN2B^2A(CT)^ mice and the Tg-GluN2D^2B(CT)^ mice demonstrated significantly less freezing than the wild-type mice, whereas the Tg-GluN2A^2B(CT)^ mice and Tg-GluN2B froze significantly more than the wild-type mice. *p<0.05, **p<1.0×10^−6^, *** p = 0.0007. (**C**) The Tg-GluN2A^2B(CT)^ mice showed quicker fear extinction than the wild-type mice in the contextual fear extinction paradigm *p<0.05, **p<0.01. (**D**) The Tg-GluN2A^2B(CT)^ mice showed quicker fear extinction to the CS than the wild-type mice in the contextual fear extinction paradigm *p<0.05, **p<0.01, ***p<0.001.

To investigate the emotional memory in the chimeric transgenic mice, we tested the mice in a contextual fear conditioning task. This type of fear conditioning is hippocampal-dependent and is often used to test short-term (one-hour) and long-term (one-day) time points. In the training session, all of the mice displayed similar freezing responses immediately after the shock was delivered (Figure 3A) (Wt: n = 13, 26.92±6.126%; Tg-GluN2A: n = 15, 25.75±3.146%; Tg-GluN2B: n = 30.00±3.637%; Tg-GluN2A^2B(CT)^: n = 13, 34.61±5.155%; Tg-GluN2B^2A(CT)^: n = 12, 25.00±7.812%; Tg-GluN2D^2B(CT)^: n = 13, 23.18±7.045%). At the one-hour retention session, the wild-type mice, the Tg-GluN2A, Tg-GluN2B^2A(CT)^ and Tg-GluN2D^2B(CT)^ mice all displayed similar freezing responses when they were returned to the shock chamber in the absence of footshock (Wt: n = 13, 26.28±4.444%; Tg-GluN2A: n = 11, 32.22±4.789%; Tg-GluN2B^2A(CT)^: n = 10, 25.28±6.286%; Tg-GluN2D^2B(CT)^: n = 13, 37.51±5.415%). Interestingly, both the Tg-GluN2B and the Tg-GluN2A^2B(CT)^ mice spent significantly more time freezing than the wild-type mice (Tg-GluN2B: n = 7, 45.06±2.823%; Tg-GluN2A^2B(CTR)^: n = 13, 52.56±3.672%; *F*(6, 61)  = 4.98, p = 0.0007). This suggests that Tg- GluN2A^2B(CT)^ mice, similar to Tg-GluN2B, exhibited enhanced 1-hr contextual fear memory.

At the one-day retention session, the Tg-GluN2B mice still displayed significantly more freezing than the wild-type mice as previously reported (Figure 3A) (n = 8, 55.94±4.911%, p = 0.039), suggesting greater long-term contextual fear memory in these mice. However, Tg-GluN2A^2B(CT)^, Tg-GluN2B^2A(CT)^ and Tg-GluN2D^2B(CT)^ mice displayed similar freezing responses as those of the wild-type mice, indicating that all of these chimeric transgenic mice have the normal 1-day hippocampal-dependent contextual fear memories. (Figure 3A) (Tg-GluN2A^2B(CT)^: n = 13, 44.87±4.779%; Tg-GluN2B^2A(CT)^: n = 12, 41.91±7.003%; Tg-GluN2D^2B(CT)^: n = 12, 47.92±7.050%). On the contrary, the Tg-GluN2A mice demonstrated reduced freezing responses during the contextual recall (Wt: n = 14, 43.65±4.382%; Tg-GluN2A: n = 13, 23.54±3.811%, *F*(5, 66)  = 3.65, p = 0.005), suggesting the deficit in converting short-term contextual fear memory into long-term contextual fear memory due to expression of GluN2A.

### Tg-GluN2B^2A(CT)^ and Tg-GluN2D^2B(CT)^ mice showed significant impairments in long-term cued fear memory, whereas Tg-GluN2A^2B(CT)^ showed enhanced memory

To assess whether and how hippocampal-independent forms of memories are affected by the N-terminal and C-terminal domain properties, we used a new cohort of the mice and tested them in cued fear conditioning task which required the mouse to associate an unconditioned stimulus (a shock) with a conditioned stimulus (a tone). In the cued fear conditioning paradigm, all of the mice exhibited little pre-tone freezing responses during the retention tests as they entered a novel chamber (Figure 3B) (Wt: n = 10, 2.22±0.997%; Tg-GluN2A: n = 15, 6.17±1.734%; Tg-GluN2B: n = 7, 6.17±1.251%; Tg-GluN2A^2B(CT)^: n = 11, 2.02±0.758%; Tg-GluN2B^2A(CT)^: n = 11, 1.26±0.576%; Tg-GluN2D^2B(CT)^: n = 12, 3.47±1.373%). Upon the recall tone, all five types of transgenic mice exhibited significant amounts of freezing responses at the one hour retention session, comparable to that of wild-type mice (Wt: n = 10, 71.67±4.843%; Tg-GluN2A: n = 18, 58.08±4.710%; Tg-GluN2B: n = 7, 62.08±6.801%; Tg-GluN2A^2B(CT)^: n = 10, 67.22±4.83%; Tg-GluN2B^2A(CT)^: n = 10, 60.00±4.833%; Tg-GluN2D^2B(CT)^: n = 9, 61.11±4.856%). This shows that the transgenic mouse lines have normal short-term hippocampal-independent emotional memory and all are able to form an association between the tone (CS) and the shock (US).

For one-day cued fear memory retention tests, a second cohort of similarly trained mice was placed into a novel enclosure and an identical tone to the training tone was presented. Interestingly, the Tg-GluN2A mice, the Tg-GluN2B^2A(CT)^ mice and the Tg-GluN2D^2B(CT)^ mice demonstrated significantly less freezing than the wild-type mice (Figure 3B) (Wt: n = 9, 55.02±3.030%; Tg-GluN2A: n = 10,14.75±5.189%; Tg-GluN2B^2A(CT)^: n = 10, 14.72±8.477%; Tg-GluN2D^2B(CT)^: n = 12, 16.67±5.772%; *F*(5, 56)  = 27.03, p<1.0×10^−6^). The Tg-GluN2A^2B(CT)^ mice froze significantly more than the wild-type mice (n = 13, 71.58±2.727%). Consistent with the previous studies (Tang et al, 1999), the Tg-GluN2B mice also showed enhanced cued memory over the wild-type mice (Tg-GluN2B: n = 8, 65.75±5.918%, p<0.05). These data demonstrate that the Tg-GluN2A, Tg-GluN2B^2A(CT)^ and Tg-GluN2D^2B(CT)^ mice have impaired long-term hippocampal-independent fear memory, whereas the Tg-GluN2A^2B(CT)^ and Tg-GluN2B mice exhibited similarly enhanced long-term cued fear memories.

### Enhanced cued fear extinction in Tg-GluN2A^2B(CT)^ mice over the wild-type mice

Fear extinction has been widely used as a test for assessing flexible learning behaviors. The extinction of learned fear requires the formation of new flexible relations, instead of forgetting or erasing the established fear memories [25]. Because the Tg-GluN2A, Tg-GluN2B^2A(CT)^, and Tg-GluN2D^2B(CT)^ mice were impaired in the one-day retention session, they were not used for the fear extinction experiment. Instead, we focused our investigation of this form of learning on the Tg-GluN2A^2B(CT)^ mice, the Tg-GluN2B mice, as well as the wild-type mice.

In this fear extinction task, we used a five trial extinction paradigm in which the animals were repeatedly exposed to the training chamber (contextual extinction) or the tone in a novel context (cued extinction) without the delivery of the shock in either context. We first tested the mice in the contextual fear extinction paradigm. We found that the Tg-GluN2B mice initially showed significantly more freezing than the wild-type mice and the Tg-GluN2A^2B(CT)^ mice 24 hours after the training session (Figure 3C) (1: Wt: n = 15, 40.19±3.852%; Tg-GluN2B: n = 8, 55.94±4.911%; Tg-GluN2A^2B(CT)^: n = 13, 44.87±4.779%). Interestingly, over the extinction trials both the Tg-GluN2B and the Tg-GluN2A^2B(CT)^ mice significantly decreased their freezing responses as early as the second session 2 hours later in comparison to the first trial (2: Tg-GluN2B: 38.29±5.161%; p = 0.014; Tg-GluN2A^2B(CT)^: 23.29±4.409%; p = 0.002), whereas the wild-type mice did not significantly decrease their freezing response from the first to the second exposure session. It is noted that the Tg-GluN2A^2B(CT)^ mice exhibited significantly less freezing, as determined by ANOVA analysis, than both the Tg-GluN2B mice and their wild-type littermates (Wt: 38.70±5.161%; *F*(2, 33)  = 3.56, p = 0.04). All groups of mice spent significantly less time freezing in the third exposure than the second exposure (3: Wt: 27.41±5.034%, p = 0.01; Tg-GluN2B: 29.12±4.294%, p = 0.03; Tg-GluN2A^2B(CT)^: 10.26±2.632%, p = 0.007). The mice continued to decrease their freezing responses in the fourth (4: Wt: 25.74±2.125%; Tg-GluN2B: 12.69±2.576%; Tg-GluN2A^2B(CT)^: 10.47±2.816%, p = 0.002) and fifth exposures (5: Wt: 21.48±3.325%; Tg-GluN2B: 6.57±1.300%; Tg-GluN2A^2B(CT)^: 9.19±2.276%). ANOVA analysis indicated that in both the fourth and fifth exposures, the wild-type mice had significantly higher freezing responses than both the Tg-GluN2B and Tg-GluN2A^2B(CT)^ mice (4: *F*(2, 33)  = 11.91, p = 0.0001; 5: *F*(2, 33)  = 8.08, p = 0.001). These data demonstrate that the Tg-GluN2B mice and the Tg-GluN2A^2B(CT)^ mice had better hippocampal-dependent fear flexibility learning ability than the wild-type mice.

Next, we exposed the mice to the tone in a novel environment for cued fear extinction learning. The Tg-GluN2A^2B(CT)^ mice, similar to the Tg-GluN2B mice, showed significantly faster cued fear extinction than the wild-type mice (Figure 3D). In the first exposure to the tone 24 hours after training, the Tg-GluN2B and Tg-GluN2A^2B(CT)^ mice showed significantly higher freezing responses than the wild-type mice (1: Wt: n = 12, 58.10±3.323%; Tg-GluN2B: n = 8, 70.87±5.017%; Tg-GluN2A^2B(CT)^: n = 13, 71.58±2.727%, *F*(2, 30)  = 4.97, p = 0.01). At the second exposure, two hours after the first exposure, the Tg-GluN2B mice and the Tg-GluN2A^2B(CT)^ mice significantly reduced their freezing responses to the presentation of the tone, whereas the wild-type mice did not (2: Wt: 57.41±4.436%; Tg-GluN2B: 41.46±2.836%, p = 0.002; Tg-GluN2A^2B(CT)^: 48.71±5.34%, p = 0.003), again suggesting the faster fear extinction in these transgenic mice. Remarkably, both the Tg-GluN2B and Tg-GluN2A^2B(CT)^ mice further decreased freezing from the second to the third exposures as well (3: Wt: 49.54±4.323%; Tg-GluN2B: 20.14±1.278%, p = 0.0004; Tg-GluN2A^2B(CT)^: 35.04±4.958%, p = 0.0005). The wild-type mice spent significantly more time freezing in the third exposure than the Tg-GluN2B and Tg-GluN2A^2B(CT)^ mice (*F*(2, 30)  = 9.87, p = 0.0005). The Tg-GluN2B mice and the Tg-GluN2A^2B(CT)^ mice continued to decrease their freezing responses in the fourth (4: Wt: 45.14±4.340%; Tg-GluN2B: 7.75±1.175%; Tg-GluN2A^2B(CT)^: 30.77±4.118%) and fifth exposures (5: Wt: 42.82±4.972%; Tg-GluN2B: 5.34±0.939%; Tg-GluN2A^2B(CT)^: 29.27±4.342%). It is worth noting that the Tg-GluN2B had the faster extinction learning. ANOVA analysis revealed that the Tg-GluN2B mice demonstrated significantly less freezing than the Tg-GluN2A^2B(CT)^ mice and the wild-type mice in both the fourth (*F*(2, 30)  = 19.32, p = 4.0×10^−6^) and fifth exposures (*F*(2, 30)  = 16.22, p = 1.7×10^−5^).

### Basic electrophysiological properties in the chimeric GluN2 mice

GluN2A and GluN2B subunits' contribution to synaptic plasticity has been intensely investigated in the CA1 region using both pharmacological and genetic methods [26]–[29]. We took advantage of the existing knowledge in the literature and investigated and compared how various chimeric transgenic overexpressions would affect the bidirectional control of synaptic plasticity in the CA1 region. To investigate the basic electrophysiological properties in the hippocampus of the Tg-GluN2A^2B(CT)^, Tg-GluN2B^2A(CT)^, and Tg-GluN2D^2B(CT)^ mice, we recorded from the CA1 Schaffer collaterals of the mouse hippocampus. We found the input-output properties (Figure S2A), as well as the paired pulse facilitation (Figure S2B) from each genotype were similar to those of the wild-type controls, thereby demonstrating normal presynaptic function and basal transmissions in these transgenic mice. We then systematically measured the long-term potentiation (LTP) and long-term depression (LTD) in the CA1 slices from each mouse line.

### Enhanced 10 Hz induced LTP observed in the Tg-GluN2A^2B(CT)^ CA1 region

**Figure 4:**
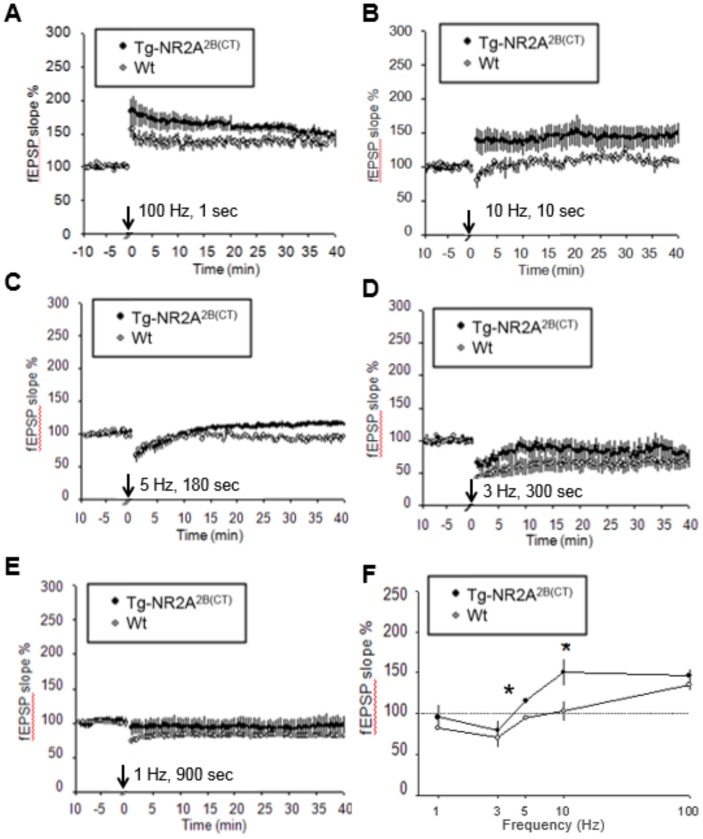
Enhanced LTP in the Tg-GluN2A^2B(CT)^ mouse hippocampal slices. A. Slightly enhanced LTP seen in the Tg-GluN2A^2B(CT)^ mice with a 1 s 100 Hz stimulation. B. Significantly enhanced LTP was seen in the Tg-GluN2A^2B(CT)^ mice when a 10 Hz stimulation was applied from 10 s. C–E. No changes in LTD were seen in the 5 Hz, 3 Hz, or 1 Hz stimulation protocols. F. A summary plot of the % change in fEPSP slope versus the frequencies.

We first performed LTP and LTD studies on the Tg-GluN2A^2B(CT)^ mice. In the Tg-GluN2A^2B(CT)^ mice, LTP can be readily induced by 100 Hz stimulation (Figure 4A) (Wt: n = 6/3(# of slices/# of animals), 135.2±7.6%; Tg-GluN2A^2B(CT)^: n = 7/4, 146.5±8.7%). Interestingly, a significant increase in LTP was observed in the transgenic mice, compared to that of wild-type slices, in response to the 10 Hz frequency stimulation (Figure 4B) (Wt: n = 7/4, 103.2±13.0%; Tg-GluN2A^2B(CT)^: n = 5/3, 150.1±17.0%). Additionally, a significant difference was further observed at 5 Hz stimulation (Figure 4C) (Wt: n = 4/3, 94.4±1.8%; Tg-GluN2A^2B(CT)^: n = 6/4, 115.5±3.9%). There is no statistical difference at the 3 Hz stimulation (Figure 4D) (Wt: n = 7/3, 70.3±11.3%; Tg-GluN2A^2B(CT)^: n = 5/3, 79.2±13.3%) or 1 Hz stimulation (Figure 4E) (Wt: n = 7/5, 82.4±1.6%; Tg-GluN2A^2B(CT)^: n = 6/3, 95.4±15.6%). Overall, we found that the Tg-GluN2A^2B(CT)^ mice show little difference in the LTD, except at 5 Hz, but show significantly enhanced LTP around at 10 Hz frequency (Figure 4F). These data indicate that the GluN2A^2B(CT)^ overexpression produced synaptic changes that were more similar to that of GluN2B overexpression in the transgenic mice and rats [14], [16], [18].

### Enhanced 10 Hz LTP and diminished 1–3 Hz LTD in the Tg-GluN2B^2A(CT)^ CA1 region

**Figure 5:**
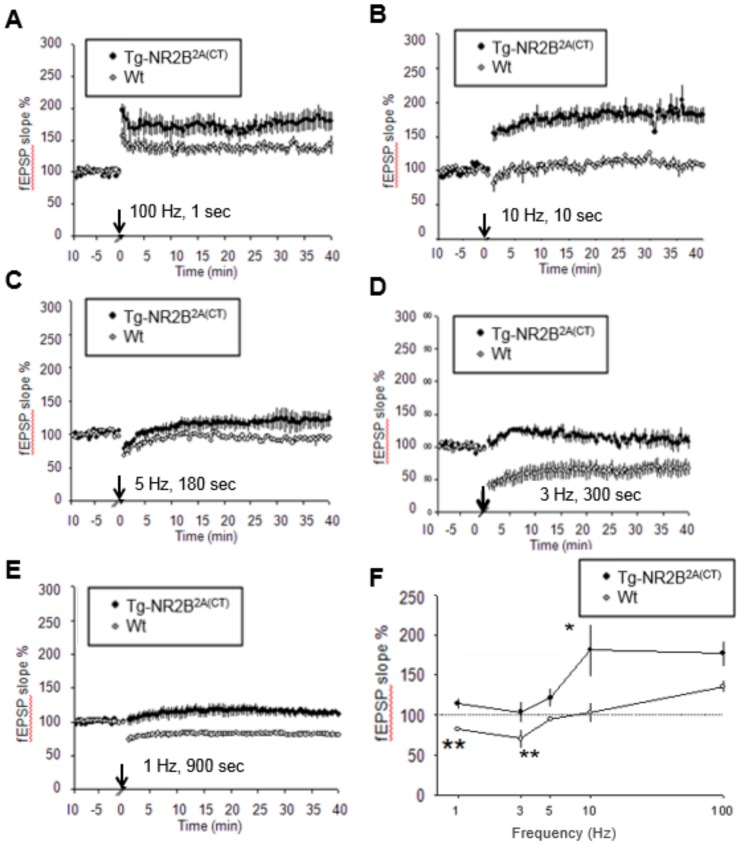
Enhanced LTP and diminished LTD in the Tg-GluN2B^2A(CT)^ mouse hippocampal slices. (**A**) A slight increase in LTP was seen in the 100 Hz stimulation protocol in the Tg-GluN2B^2A(CT)^ mice. (**B**) When a 10 Hz stimulation was applied for 10 s a significant increase in LTP was seen in the Tg-GluN2B^2A(CT)^ mice over their wild-type littermates. (**C**) LTD was diminished at 5 Hz stimulation in the Tg-GluN2B^2A(CT)^ mice. (**D**) LTD was significantly diminished at the 3 Hz stimulation protocol. (**E**) At the 1 Hz stimulation, the Tg-GluN2B^2A(CT)^ mice show significantly diminished LTD. (**F**) A summary plot of the % change in fEPSP slope versus the frequencies.

We then measured synaptic plasticity in the Tg-GluN2B^2A(CT)^ CA1 slices. Overexpression of GluN2B^2A(CT)^ significantly increased LTP versus their wild-type littermates at both 100 Hz (Figure 5B) (n = 13/6, 176.6±16.2%; Figure 5A) and 10 Hz (n = 5/3, 180.7±33.0%) frequencies. Interestingly, while 10 Hz response did not differ, LTD was also significantly impaired as compared to the wild-type hippocampal slices at 5 Hz (n = 4/2, 121.6±2.2%; Figure 5C), 3 Hz (n = 6/3, 103.6±13.8%; Figure 5D) and 1 Hz (n = 21/13, 114.2±6.6%; Figure 5E). This shows that although the Tg-GluN2B^2A(CT)^ mice have significantly increased LTP, they also have significantly blocked 1 Hz and 3 Hz induced LTD (summarized in Figure 5F). This decrease in LTD in Tg-GluN2B^2A(CT)^ slices was more similar to that seen in the Tg-GluN2A mice [23].

### Impaired 5 Hz responses in the Tg-GluN2D^2B(CT)^ CA1 region

**Figure 6:**
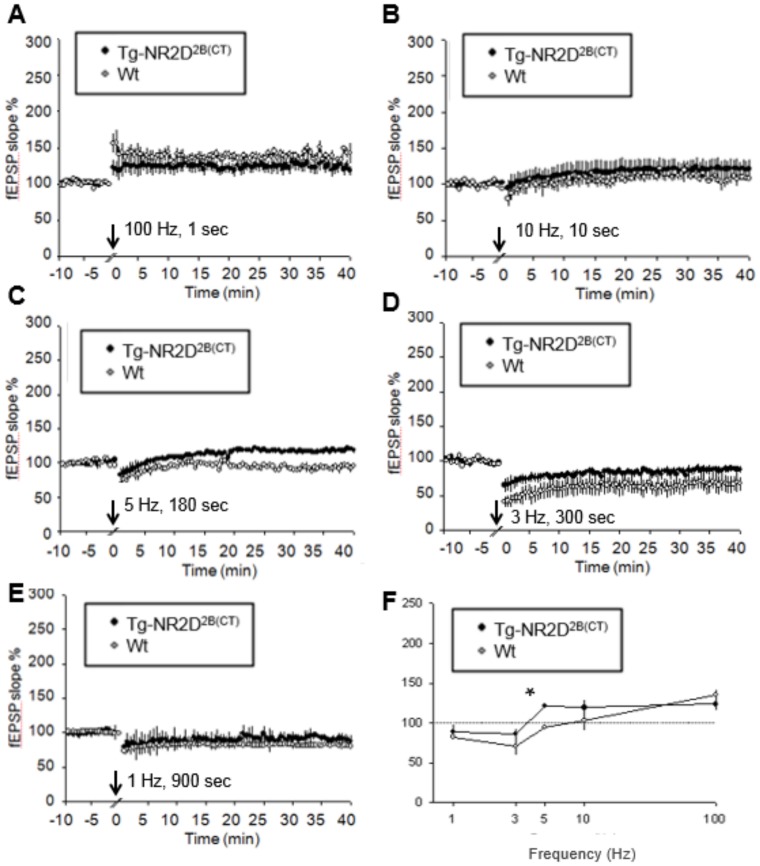
Diminished LTD at the 5 Hz range in the Tg-GluN2D^2B(CT)^ mouse hippocampal slices. (**A**) No changes from the wild-type hippocampal slices were seen in the LTP of the Tg-GluN2D^2B(CT)^ mouse hippocampal slices when a 100 Hz stimulation was applied. (**B**) When a 10 Hz stimulation was applied for 10 s there, again was no significant change in LTP observed in the Tg-GluN2D^2B(CT)^ mice over their wild-type littermates. (**C**) LTD was diminished at 5 Hz stimulation in the Tg-GluN2D^2B(CT)^ mice. (**D**) LTD was not significantly diminished at the 3 Hz stimulation protocol. (**E**) At the 1 Hz stimulation, the Tg-GluN2D^2B(CT)^ mice showed no significant differences in LTD. (**F**) A summary plot of the % change in fEPSP slope versus the frequencies.

Finally, we examined the effects of GluN2D^2B(CT)^ overexpression on CA1 plasticity. Since GluN2D has very weak magnesium dependency but much greater opening duration, it would lead to significantly more Ca^2+^ influx into the postsynaptic sites. We performed LTP and LTD measurements on the Tg-GluN2D^2B(CT)^ hippocampal slices. Interestingly, we found no differences at either the 100 Hz (Figure 6A) (n = 8/6, 128±8.6%) or the 10 Hz frequency between the transgenic and control littermates (Figure 6B) (n = 6/3, 125.5±12.6%). However, at the 5 Hz frequency, a small, but significant LTP was observed in the transgenic slices, in comparison to the LTD induced in the slices from the control littermates (Figure 6C) (n = 5/4, 123.5±3.6%). However, there were no significant differences observed in LTD at 1 Hz stimulation (Figure 6E) (n = 8/4, 88.6±7.6%), 3 Hz (Figure 6D) (n = 7/5, 87.6±12.4%). The summary graphs show the overall similarities between the Tg-GluN2D^2B(CT)^ mice and their wild-type counterparts, except in its 5 Hz frequency response (Figure 6F).

## Discussion

The NMDA receptor is widely known as the key coincidence-detector at central synapses to implement Hebb's learning rule. The channel opening-duration and the level of membrane depolarization, determines the amount of Ca^2+^ that influxes into the cell [30], [31]. In this study, we have identified the critical molecular motifs of the GluN2 subunits essential for achieving learning and memory enhancement in the adult mouse brain. By systematically analyzing three chimeric GluN2 transgenic mice together with Tg-GluN2A and Tg-GluN2B mice, we have tested two major hypotheses, namely, synaptic coincidence-detection/calcium influx hypothesis vs. GluN2B C-terminal intracellular signaling hypothesis in gating memory enhancement. Our experiments have revealed several novel insights into the relationships between GluN2 subunit motifs, synaptic plasticity, and memory enhancement.

The “synaptic coincidence-detection” hypothesis reflects the predominant view in the field as the rate-limiting factor in determining learning and memory capability. It posits that because the GluN2B subunit makes the channel opening duration longer than that of the GluN2A subunit, GluN2B overexpression allows a greater coincidence detection window, thereby leading to superior memory functions [4]. Because the N-terminal and transmembrane domains of the GluN2 subunits are known to be crucial for controlling voltage-gating and ion (Ca^2+^) influx duration, we replaced the GluN2B N-terminal domains with either GluN2A or GluN2D while retaining its wild-type C-terminal intracellular domain. As such, these two chimeric GluN2 subunits possessed the GluN2B intracellular signaling capability but with the other key properties such as the shorter opening duration and the voltage-dependency from the GluN2A and GluN2D, respectively.

As predicted, because GluN2D has greatly reduced Mg^2+^ dependency, which renders synaptic coincidence-detection ineffective, GluN2D^2B(CT)^ transgenic mice indeed exhibited memory deficits in novel object recognition (in both the short-term and long-term form) and long-term cued fear conditioning memory (although the contextual fear memory seemed to be normal). These observations have provided evidence that synaptic coincidence-detection is necessary for producing memory enhancement via the GluN2B intracellular signaling cascades. Without proper magnesium dependent voltage gating, the presence of the overexpressed GluN2B domain from the chimeric GluN2D^2B(CTD)^ subunit still could not produce optimal synaptic changes for memory enhancement.

On the other hand, we were quite surprised that the Tg-GluN2A^2B(CT)^ transgenic mice exhibited a very similar memory enhancement phenotype to those of the Tg-GluN2B mice. The different channel opening-durations derived from GluN2A and GluN2B subunits' N-terminal and transmembrane domains are not the most critical factor in determining the memory enhancement, as long as the GluN2B C-terminal domain is transducing the signaling. It is important to note here that Punnakkal et al. found little differences in the whole cell currents of similar chimeric constructs, with only a slight decrease in the peak amplitude of a similar GluN2AB construct from that of the GluN2A wildtype subunit [32]. No changes in the peak amplitude of a similar GluN2BA construct over that of the GluN2B wildtype subunit. Additionally, deactivation times remained unchanged between the wildtype and chimeric receptors. Importantly, they also concluded that the peak opening probability appeared to be determined by the GluN2 N-terminal domain [32]. Therefore, these genetic experiments have shown that Mg^2+^-dependent coincidence-detection function, but not necessarily the opening-duration difference between GluN2A and GluN2B, is prerequisite for achieving learning and memory enhancement in the adult brain.

**Figure 7:**
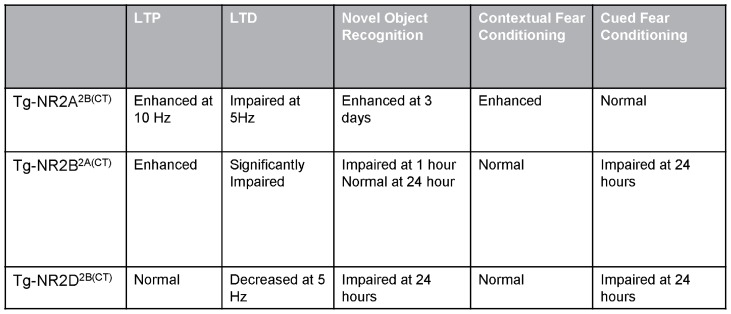
Summary of LTP, LTD and behavioral tasks results. The Tg-NRA^2B(CT)^ mice had enhanced LTP, as well as enhanced long-term recognition memory and contextual fear conditioning. The Tg-GluN2B^2A(CT)^ mice have significantly impaired LTD resulting in impaired short-term recognition memory and impaired long-term cued fear conditioning. The Tg-GluN2D^2B(CT)^ mice have decreased LTD at 5 Hz and impaired long-term recognition memory and long-term cued fear memory.

Interestingly our present study has provided clear evidence supporting the second hypothesis that is known as the “GluN2B intracellular domain” hypothesis [4], [33]. Two separate pieces of evidence came from our behavioral analyses of the Tg-GluN2B^2A(CT)^ and Tg-GluN2A^2B(CT)^ transgenic mice. First, we found that the Tg-GluN2A^2B(CT)^ mice had enhanced object recognition memory and emotional memory. These phenotypes are very similar to those of the Tg-GluN2B mice (and also Tg-GluN2B rats) (Figure 7). On the contrary, when the C-terminal domain of the GluN2B subunit was replaced by that of the GluN2A subunit, as we did in Tg-GluN2B^2A(CT)^ mice, this swap led to profound memory deficits in novel object recognition test and long-term cued fear memories. These memory deficits mirrored those of Tg-GluN2A mice [23]. These subunits-swap experiments, by extending to learning and memory enhancement, are consistent with other reports that the intracellular domains of the GluN2 subunits play critical roles in mediating different functions, such as synaptic localization, clustering, signal transduction, and behaviors [22], [33]–[39]. Therefore, our studies suggest that both “synaptic coincidence-detection” hypotheses and “GluN2B intracellular signaling” hypothesis are mutually complementary in term of explaining the molecular determinants for memory enhancement.

Two additional conceptual insights have also been obtained on how different molecular motifs of the overexpressed GluN2 subunits regulate the levels and degrees of LTP or LTD over a wide range of stimulation frequencies. Despite multiple pharmacological and knockout approaches to analyzing GluN2A and GluN2B on regulating LTP and LTD [40], few were done under the context of examining its relationship with cognitive enhancement [16], [18], [41]. Here, we consistently found that GluN2B and GluN2A^2B(CT)^ overexpression enhanced LTP in the range of 10 Hz and/or 100 Hz range without significantly affecting 1 Hz or 3 Hz LTD. It is noteworthy to point out that that Tg-GluN2A^2B(CT)^ seemed to produce larger 100 Hz LTP in the initial 20∼30 minutes range than that of wild-type slices, but become indistinguishable by the 40-minutes time points. Interestingly, the Tg-GluN2B overexpression tended to produce much larger 100 Hz induced LTP in comparison to the wild-type mice well beyond 60 minutes [16]. This indicates the longer time duration of channel opening (thereby more calcium influx) via the GluN2B N-terminal and pore does make larger and more stable LTP in response to 100 Hz stimulation [42], [43]. However, at the 10 Hz stimulation range, the amount of calcium influx via the GluN2A N-terminal and pore domains (coupled to GluN2B C-terminal region) can produce the similarly larger LTP in the Tg-GluN2A^2B(CT)^ mice as that of the Tg-GluN2B mice in comparison to that of the wild-type controls. This 10 Hz stimulation frequency can be particularly interesting because we have observed that fear conditioning-induced firing increase in CA1 pyramidal cells is mostly in the range of 5∼30 Hz [6], [44]. This behaviorally relevant frequency range deserves special investigation for memory enhancement in future experiments both in the hippocampus and other brain regions such as the prefrontal cortex and amygdala. In addition, contrary to LTD produced by 5 Hz stimulation in the wild-type slices, Tg-GluN2D^2B(CT)^ slices exhibited a significant switch to LTP. Taken together, these findings have provided additional support for the notion that the GluN2B C-terminal domain plays a key role in regulating LTP [26], [45]–[48], and more importantly, our study has further defined, for the first time, its essential link to memory enhancement.

While it is evident that the C-terminal of the GluN2B subunit plays a crucial role in producing synaptic potentiation, we found that Tg-GluN2B^2A(CT)^ mice had larger, more robust, LTP not only at 10 or 100 Hz. Intriguingly, such a swap also promoted an overall shift toward potentiation even in response to lower frequencies. As a result, the ability to produce LTD at 1–3 Hz frequency range is greatly impaired in Tg-GluN2B^2A(CT)^ slices. These findings show that longer opening duration achieved by the overexpressed GluN2B N-terminal and pore domains, but coupled to the GluN2A intracellular signaling cascade, brings a greater potentiation but at the cost of losing synaptic depression capacity. This is in stark contrast with the normal LTD in Tg-GluN2B or Tg-GluN2A^2B(CT)^ in response to 1 or 3 Hz stimulation. This strongly suggests that increased calcium influx (via the GluN2B N-terminal and core domains) is useful to produce bigger LTP, but its effect on 1 Hz LTD critically depends on whether the downstream signaling cascade is mediated by the GluN2A C-terminal tail or GluN2B C-terminal tail. In other words, under such circumstance, the presence of chimeric GluN2A C-terminal domain, but not chimeric GluN2B C-terminal domain, can override LTD. This novel insight adds to the notion that GluN2A may have a general ability to drive toward LTP [49]–[54].

In addition, by taking advantage of the correlational analysis between synaptic changes and memory performances, our present study has uncovered two detailed insights into the memory enhancement strategy: first, bigger LTP would lead to better learning and memory, however, only if the LTD ability remains intact. This is supported by the observation that bigger CA1 LTP is associated with better memory in Tg-GluN2B and Tg-GluN2A^2B(CT)^ mice while their LTD was not altered. Second, if LTP enhancement results in overriding or diminishing LTD capacity, such as those observed in Tg-GluN2B^2A(CT)^, it would also lead to memory deficits. The Tg-GluN2B^2A(CT)^ phenotypes are more similar to the knockout of PSD-95 which also leads to larger LTP, lack of 1 Hz LTD, and memory deficits (i.e. [55], [56]). Our recent characterization of Tg-GluN2A mice showed that overexpression of GluN2A results in no change in 100 Hz LTP or 1 Hz, but greatly impaired 3 or 5 Hz LTD. These mice also exhibited long-term memory deficits, while short-term memories remained mostly normal. Our Tg-GluN2D^2B(CT)^ mice, which also showed 5 Hz LTP responses instead of either no change or LTD, as in the wild-type mice, were also profoundly impaired in long-term memory. These observations support the “LTD-memory trace sculpting” hypothesis, that the weakening of uncorrelated synaptic connections would reduce the background “noise” while enabling the stabilization (or crystallization) of the learning-related synaptic patterns [23]. However, it is important to note that our current electrophysiological recordings were limited to the CA1 region. Given the fact that we used the CaMKII promoter to drive the transgenes, electrophysiological analyses should be extended in future experiments into other brain regions such as the amygdala and prefrontal cortex from which cued fear learning and fear extinction are processed. Clearly, simple correlation between CA1 synaptic plasticity and memory are likely not sufficient for counting memory enhancement and thus, any extrapolation should be only taken with great caution. In addition, little is known about how any of the artificial stimulation paradigms for producing LTP or LTD can be translated into real-time memory patterns. Recent successful decoding of real-time fear memory traces in the hippocampal CA1 from the wild-type mice and the forebrain excitatory neuron-specific NMDA receptor inducible knockout mice, have revealed many fundamental insights how the NMDA receptors regulate real-time memory code and memory engrams [6], [44]. It would be of great interest to use such brain decoding technologies to investigate the various transgenic mice described here.

In summary, our above experiments have identified the key molecular and genetic determinants that would be necessary and sufficient for achieving superior learning and memory ability in the adult brain. Although transgenetic methods are unlikely to be used for human clinical settings, the C-terminal region of the GluN2B subunit contains many important sites for various molecular interaction including with CaMKII, cdk5, and Kinesin superfamily protein 17 (KIF17) [57]–[60]. Indeed, manipulations of cdk5 and KIF17 which result in upregulation of GluN2B also resulted in memory enhancement [61], [62]. More recently, researchers have taken a novel, dietary approach to up-regulate GluN2B expression in the brain via elevating brain magnesium [63]. They showed that the compound, magnesium threonate, can cross the blood brain barrier efficiently and boost GluN2B expression in the neurons, and subsequent memory improvement in both aging and wild-type mice [64], [65]. This compound is currently under clinical trials [66]. Therefore, it is conceivable that knowledge gained from the present study will be valuable to the current efforts in developing and optimizing memory enhancement strategies.

## Methods

### Production of Transgenic Mice

We have produced three chimeric GluN2 subunit constructs for the present study. In the first two constructs, the N-terminal and transmembrane domains of GluN2A or GluN2D subunit were fused with the C-terminal domain from the GluN2B subunit, termed GluN2A^2B(CTR)^ and GluN2D^2A(CTR)^, respectively. In the third construct, we also fused the N-terminal and transmembrane domain of GluN2B subunit with the C-terminal domain from the GluN2A subunit, termed GluN2B^2A(CTR)^ (see Figure 1A). The fusion site was located near the end of the fourth transmembrane domain, just before the C-terminal domains begin. For making the constructs, we first introduced a point mutation to create a unique Aat II cutting site for the fusing of the given C-terminal domain (Figure 1B). Upon successful ligation, the point mutation was mutated back to its original sequence. These chimeric transgene constructs were driven by the forebrain-specific αCaMKII promoter for targeting their expression to the excitatory neurons in the forebrain regions such as the cortex and hippocampus.

The chimeric constructs were created by first introducing a point-mutation at the site to create a unique Aat II cutting site for swapping the NT and CT domains. Upon successful ligation the point mutation was swapped back to the original sequence. The modified subunit was targeted for forebrain expression by the CaM-kinase II (CaMKII) promoter as previously described [16], [67]. The founding line of transgenic animals was produced by pronuclear injection of a linearized chimeric transgene vector into C57BL/6J zygotes similar to previously described [16], [68]. A total of seven independent mouse founder lines (three lines for GluN2A^2B(CTR)^ termed “Tg-GluN2A^2B(CT)^” mice, two lines for GluN2B ^2A(CTR)^ termed “Tg-GluN2B^2A(CT)^” mice, and two lines for the GluN2D^2B(CTR)^ termed “Tg-GluN2D^2B(CT)^” mice). All these lines gave successful germline transmissions. The genotypes of the transgenic mice were determined by PCR analysis of a tail biopsy. The transgene was detected using the SV40 poly(A) sequence, as previously described [16], [18], [41]. Southern blotting was used to confirm the transgene integration in to the transgenic mouse line. Western blotting of the forebrain regions (cortex and hippocampus) was visualized with either a polyclonal GluN2A C-terminal antibody (Upstate/Millipore) or a polyclonal GluN2B C-terminal antibody (Millipore). For the present electrophysiological and behavioral experiments, we used a high expression line chosen from the Tg-GluN2A^2B(CT)^ mice, the Tg-GluN2B^2A(CT)^ mice, and the Tg-GluN2D^2B(CT)^ mice that have been crossed with C57BL/6J wildtype mice for at least 8 generations. For *in situ* hybridizations, brains from the transgenic mice and wild-type littermates were isolated and 20 µm sections were prepared using a cryostat. The slices were hybridized to the [α^35^S] oligonucleotide probe which hybridized to the untranslated artificial intron region in the transgene similarly to previously described [16], [23].

### Behavioral Experiments

Mice were maintained in a temperature and humidity controlled vivarium with a 12:12 light-dark cycle. All testing was done during the light phase with 3–5 month old animals. Mice were allowed free access to food and water, except during experimental procedures. Mice were extensively handled prior to any testing paradigm. Separate cohorts were used for each study and each recall time point unless otherwise stated. All testing procedures were conducted in sound dampened, dimly lit behavioral rooms. Experimenters were blind to the genotype of the animals. This study was carried out in strict accordance with the recommendations in the Guide for the Care and Use of Laboratory Animals of the National Institutes of Health. The protocols were approved by the Institutional Animal Care and Use Committee of the Georgia Regents University.

### Open Field

One cohort of Tg-GluN2A, Tg-GluN2B, Tg-GluN2A^2B(CT)^, Tg-GluN2B^2A(CT)^, and Tg-GluN2D^2B(CT)^ mice and their wild-type littermates were individually placed into a 50 cm L×50 cm W×25 cm H white Plexiglas open field arena. The mouse was allowed to explore for ten minutes. The time that the mouse spent in the center and periphery was determined. The periphery of the open field was considered to be the first four inches along the wall, while the center of the open field was the square inside this area [69]. Additionally, the distance traveled by the mouse was determined using Biobserve Viewer II software.

### Elevated Plus Maze

The elevated plus maze consisted of a black Plexiglas “plus” maze approximately 60 cm above the floor, with each arm measuring 30 cm in length and 10 cm wide. Two opposite arms were left open, with the other two arms being enclosed on three sides. The ambient room lighting was 75 lux. The amount of time the mice spent within the enclosed arms was recorded, as well as the amount of time the animal spent in the open arms [69]. The times were used to determine a preference index.

### Novel Object Recognition

The behavioral paradigm was the same as previously described [16], [70]. The mice were individually habituated to a 50 cm L×50 cm W×25 cm H open field apparatus for 10 minutes a day for three days. On the first testing day, the mice were placed into the open field with two identical objects for 5 minutes. The time they spent exploring each object was recorded. At the described retention time the mice were placed back into the open field arena with one of the familiar objects used in training, and one novel object, and allowed to explore for 5 minutes. The time they spent with each object was recorded and used to determine a preference index. Different groups of mice were used for the each retention session.

### Fear Conditioning

An operant chamber (25 cm L×25 cm W×38 cm H) equipped with activity monitors and camera was used. The flooring was a 24 bar shock grid with a speaker, shock generator, and photo-beam scanner (MedAssociates). The chamber was located in a sound damping isolation box. The apparatus was thoroughly cleaned with 70% ethanol between mice to avoid any olfactory cues. Freezing was monitored by the software and confirmed by the experimenter.

Testing procedures were similar to those previously described [16], [71], [72]. Animals were habituated to the testing environment for 5 minutes one day before testing. On the day of training the mice were placed into the chamber and allowed to explore for 5 minutes. Then the mice were exposed to a conditioned stimulus (CS, 85 dB tone at 2800 Hz), with the unconditioned stimulus (US, a scrambled foot shock at 0.75 mA) occurring the last 2 seconds of the CS. The mice were allowed to stay in the chamber for 30 seconds after the CS/US pairing to monitor immediate freezing.

To test the contextual freezing exhibited by the animal, at the described time (1 hour or 24 hours) the trained mice were placed back into the shock chamber for 5 minutes while their freezing response was monitored. The mice were then placed into a novel chamber and monitored for their freezing response (pre-tone) for 3 minutes before the onset of the CS tone for 3 minutes. During the tone the animal's freezing response was monitored to test the cued fear retention.

Freezing was judged as the complete immobility of the animal, except for movement necessary for respiration. The mice were then returned to their home cage for either 1 hour or 24 hours. At the described time the mice were returned to the chamber for measurement of the contextual freezing. The mice were then placed in a novel chamber and the tone was delivered for 3 min, during which their cued freezing response was monitored. To test the fear extinction of the animals the same recall testing paradigm was repeated at 2 hour intervals for four additional trials.

### Statistical analysis of behavioral data

All behavioral data are presented as mean ± SEM. Significance was determined by ANOVA analysis with Tukey-Kramer, or a Student's t-test. P values of <0.05 were considered significant.

### Hippocampal Slice Recordings

Transverse slices of the hippocampus were rapidly prepared from wild-type and Tg-GluN2A^2B(CT)^, Tg-GluN2B^2A(CT)^, and Tg-GluN2D^2B(CT)^ mouse lines (3∼6 months old) and maintained in an interface chamber at 28°C and were subfused with artificial cerebral spinal fluid (ACSF, 124 mM NaCl, 4.4 mM KCl, 2.0 mM CaCl_2_, 1.0 mM MgSO_4_, 25 mM NaCHO_3_, 1.0 mM Na_2_HPO_4_ and 10 mM glucose) and bubbled with 95% O_2_ and 5% CO_2_. Slices were kept in the recording chamber for at least two hours. A bipolar tungsten stimulating electrode was placed in the stratum radiatum in the CA1 region. A glass microelectrode (3–12 MΩ) filled with ACSF was used to measure the extracellular field potentials in the stratum radiatum. Test response elicited at 0.02 Hz. Current intensity (0.5–1.2 mA) which produced 30% of maximal response was used for studies of PPF and synaptic plasticity at different frequencies. Various interpulse intervals (20–400 msec) were used for measuring PPF. Low-frequency stimulation of (5 Hz for 3 min, 3 Hz for 300 s, or 1 Hz for 900s) was then used to produce depotentiation [73]. Long term potentiation was induced by tetanic stimulation (100 Hz for 1 s and 10 Hz stimulation for 10 s). Data are expressed as mean ± SEM. One-way ANOVA (with Duncan's multiple range test for *post hoc* comparison) and Student's t-test were used for statistical analysis. The detailed procedures were the same as described (Tang et al. 1999; Shimizu, et al. 2000; Wang et al. 2003; Wang, et al. 2008).

## Supporting Information

Figure S1 **Conformation of the integration of the transgene.** (**A**) Southern Blot analysis of Tg-GluN2(A/B), Tg-GluN2(B/A), and Tg-GluN2(D/B) mice. The numbers indicate the positive control and the copy number. (**B**) Western Blot analysis of the chimeric animals showing enhanced expression of the GluN2A C-terminal domain in the Tg-GluN2A, Tg-GluN2(B/A) mice and no enhancement of the GluN2A C-terminus in the Tg-GluN2(A/B) and Tg-GluN2(D/B) mice. (**C**) Western Blot analysis of the chimeric animals showing enhanced expression of the GluN2B C-terminal tail in the Tg-GluN2(A/B), and Tg-GluN2(D/B) mice of the expression in the wild-type mice.
(TIF)

Figure S2 **Electrophysiology of hippocampal slices.** (**A**) There were no significant differences in the basal synaptic transmission as seen in the CA3-CA1 input-output curve between the wildtype mice and the transgenic mice. (**B**). The paired-pulse facilitation was unchanged between the wildtype and the chimeric transgenic mice indicating that the presynaptic function is unchanged.
(TIF)
