# Supplementary figures and images for: Molecular and Genetic Determinants of the NMDA Receptor for Superior Learning and Memory Functions
Source: PLoS One. 2014 Oct 31;9(10):e111865. doi: 10.1371/journal.pone.0111865 (PMC4216132; doi:10.1371/journal.pone.0111865)

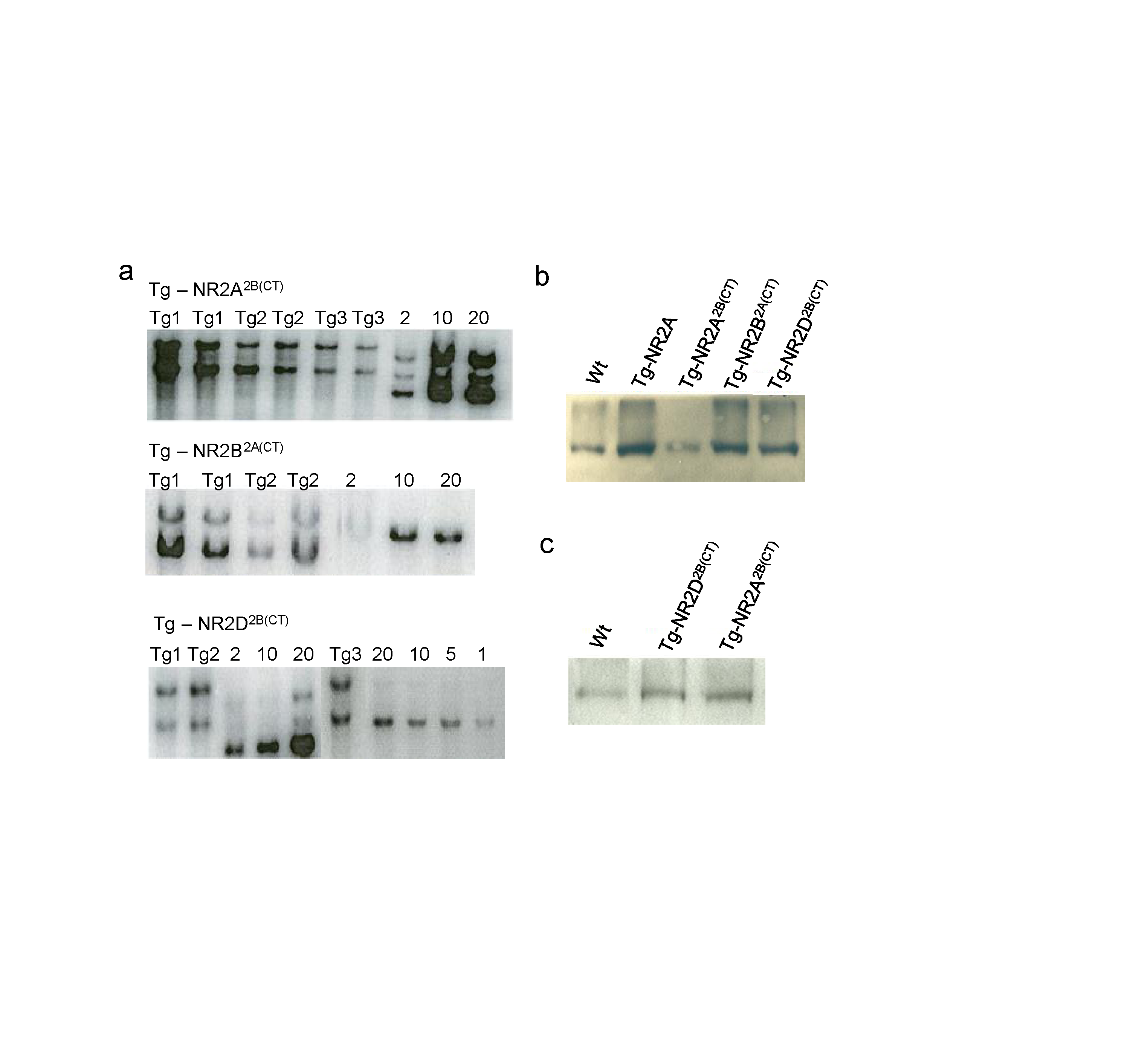

Supplement: Figure S1 — Conformation of the integration of the transgene. (A) Southern Blot analysis of Tg-GluN2(A/B), Tg-GluN2(B/A), and Tg-GluN2(D/B) mice. The numbers indicate the positive control and the copy number. (B) Western Blot analysis of the chimeric animals showing enhanced expression of the GluN2A C-terminal domain in the Tg-GluN2A, Tg-GluN2(B/A) mice and no enhancement of the GluN2A C-terminus in the Tg-GluN2(A/B) and Tg-GluN2(D/B) mice. (C) Western Blot analysis of the chimeric animals showing enhanced expression of the GluN2B C-terminal tail in the Tg-GluN2(A/B), and Tg-GluN2(D/B) mice of the expression in the wild-type mice. (TIF) [file pone.0111865.s001.tif]

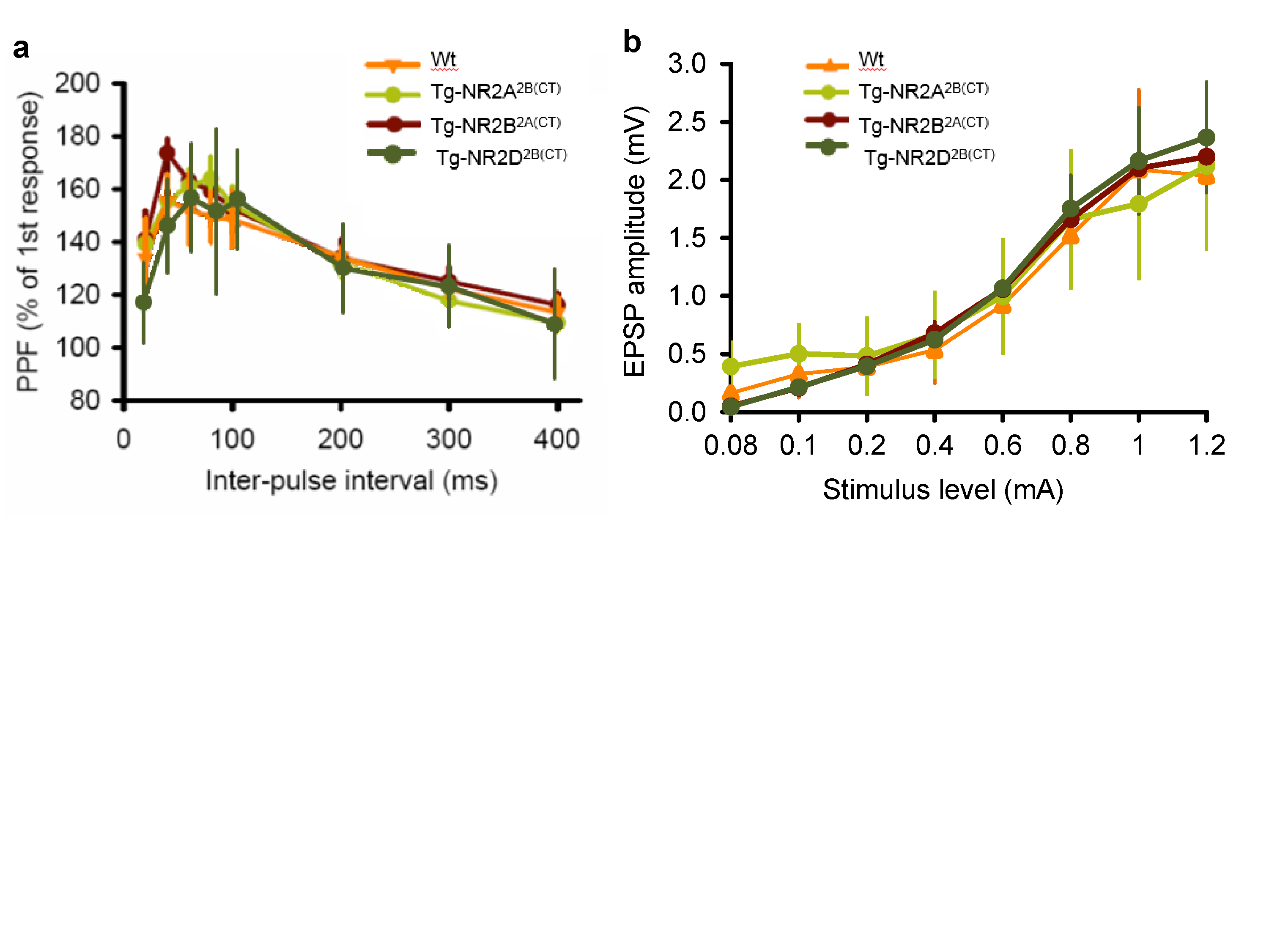

Supplement: Figure S2 — Electrophysiology of hippocampal slices. (A) There were no significant differences in the basal synaptic transmission as seen in the CA3-CA1 input-output curve between the wildtype mice and the transgenic mice. (B). The paired-pulse facilitation was unchanged between the wildtype and the chimeric transgenic mice indicating that the presynaptic function is unchanged. (TIF) [file pone.0111865.s002.tif]
